# Long noncoding RNA *MEG3* decreases the growth of head and neck squamous cell carcinoma by regulating the expression of miR‐421 and E‐cadherin

**DOI:** 10.1002/cam4.3002

**Published:** 2020-04-11

**Authors:** Yefeng Ji, Guanying Feng, Yunwen Hou, Yang Yu, Ruixia Wang, Hua Yuan

**Affiliations:** ^1^ Jiangsu Key Laboratory of Oral Diseases Nanjing Medical University Nanjing China; ^2^ Department of Oral and Maxillofacial Surgery Affiliated Hospital of Stomatology Nanjing Medical University Nanjing China

**Keywords:** epithelial‐mesenchymal transition, head and neck squamous cell carcinoma, maternally expressed 3, miR‐421

## Abstract

**Background:**

Maternally expressed 3 (*MEG3*), a long chain noncoding RNA (lncRNA), has verified its function as a suppressor in several kinds of cancers. However, the downstream mechanism of *MEG3* in regulating the molecular mechanism of epithelial‐mesenchymal transformation (EMT) in head and neck squamous cell carcinoma (HNSCC) progression demands further investigation.

**Methods:**

Quantitative real‐time polymerase chain reaction (qRT‐PCR) was used to determine the expression level of *MEG3* in HNSCC and adjacent normal tissues of 51 cases. Luciferase report assay was used to detect the correlation between miR‐421 and *MEG3*, and miR‐421 and E‐cadherin in HNSCC cell lines. Cell invasion and proliferation capacity were assessed through transwell and CCK8 assays. Scratch wound assay was used to assess cell migration capacity.

**Results:**

Firstly, this study demonstrated that the expression of *MEG3* was significantly downregulated in HNSCC compared to adjacent normal tissues. Overexpressed *MEG3* inhibited cell proliferation, migration, and invasion in vitro. Secondly, *MEG3* upregulated the expression of E‐cadherin, which was instead downregulated by miR‐421. MiR‐421 was negatively regulated by *MEG3* in HNSCC. Therefore, *MEG3* regulated EMT by sponging miR‐421 targeting E‐cadherin in HNSCC.

**Conclusions:**

This study indicated that the *MEG3*‐miR‐421‐E‐cadherin axis could be a new therapeutic target for HNSCC.

## Graphical abstract

Overexpressed MEG3 inhibits cell proliferation, migration, and invasion. MEG3 upregulated the expression of E‐cadherin, which was instead downregulated by miR‐421. MiR‐421 is negatively regulated by MEG3 in HNSCC. MEG3 therefore regulates EMT by sponging miR‐421 targeting E‐cadherin in HNSCC

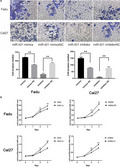

## 1 INTRODUCTION

Head and neck squamous cell carcinoma is a common malignant cancer with an estimated incidence of 53 000 new cases and 10 860 deaths in 2019 according to the cancer statistics (Surveillance and Health Services Research, American Cancer Society) in the United States, and its incidence is increasing year by year.^1^ The survival rate has not improved significantly though advanced treatments have been put into use in recent decades.^2^ Effective treatment with early diagnosis leads to a better response and survival rate.^3^, ^4^

In the past few years, it is challenging and exciting to investigate the molecular mechanism of noncoding RNAs (ncRNAs) in cancer etiology and pathophysiology. miRNA is a subclass of small ncRNAs with 21‐25 nucleotide long. Many studies have suggested the critical role of lncRNA in the cancerogenic process and its potential role as a biomarker in cancer.^5^, ^6^, ^7^ Long noncoding RNA *MEG3* (lncRNA *MEG3*) has been reported to function as a cancer suppressor gene in a variety of solid cancers.^8^, ^9^, ^10^, ^11^, ^12^, ^13^, ^14^, ^15^, ^16^ Normally, a cancer suppressor gene's product is able to inhibit cancer initiation and progression in P53 pathway, Wnt/beta‐catenin pathway, and epithelial‐mesenchymal transition (EMT).^17^ These pathways have been reported to contribute to the regulator roles of *MEG3* in a variety of cancers such as breast, meningioma, kidney, and cervical cancers.^9^, ^10^, ^11^, ^12^, ^18^ Epithelial‐mesenchymal transition (EMT) is a biological process in which epithelial cells are transformed into mesenchymal phenotypes, which trigger the dissociation of carcinoma cells from primary carcinomas, accompanied with increased invasive and migrate potential.^19^ When EMT occurs, the expression of mesenchymal markers Vimentin and ZEB2 will be upregulated, while the expression of epithelial markers E‐cadherin downregulated. The mechanism of *MEG3* regulating cell proliferation and migration in the EMT process is unclear.^20^, ^21^ Recently, it has been found that miR‐421 is able to promote cell proliferation and migration in hepatocellular carcinoma, nasopharyngeal carcinoma, and neuroblastoma.^22^, ^23^, ^24^ In this study, we hypothesized miR‐421 to regulate the EMT process targeting *MEG3* through miRanda software analysis. Also, we explored the interactions of *MEG3*, miR‐421, and E‐cadherin in head and neck squamous cell carcinoma (HNSCC) tissues and cells.

## 2 MATERIALS AND METHODS

### 2.1 HNSCC tissues

We obtained primary HNSCC tissues and adjacent normal tissues of 51 cases of patients who received radical surgery for HNSCC in Stomatological Hospital of Jiangsu Province (China) from January 2015 to June 2016. The diagnosis and stage were determined according to pathology and the cancer staging manual (seventh edition). Patients who received chemotherapy, radiotherapy, or any other medical intervention were excluded. Tissue specimens were frozen and stored in the −80°C ultra‐low freezer. Every patient signed an informed consent and the study was approved by the research ethics committee of Stomatological Hospital of Jiangsu Province.

### 2.2 Cell culture and transfection

HNSCC cell lines (Cal27, HN4, and Fadu) were purchased from ATCC (American Type Culture Collection). HN4 cells were kindly gifted by Prof. Wantao Chen (Shanghai Jiao Tong University). Human oral epithelial cell (HOEC) was purchased from Department of Oral and Maxillofacial Surgery of Wuhan University. *MEG3* overexpression and negative control lentivirus, miR‐421‐mimics/inhibitor plasmids were purchased from GeneChem Biotechnology Company. Complete growth medium was prepared with Dulbecco's Modified Eagle Medium (DMEM) (Gibco), 10% fetal bovine serum (FBS) (HyClone Laboratories) and supplemented with 2% penicillin‐streptomycin antibiotic. Fadu and Cal27 cells were cultured in complete medium in 37°C incubator with 5% CO_2_ for 24 hours. Lentivirus vector was transfected into cells. Then change the culture medium to fresh in the next 24 hours. Cells were subcultured at the densities higher than 80% confluency in 48 or 72 hours. Lipofectamine 2000 Transfection Reagent (Thermo Fisher Scientific) was used to transfect cells with plasmids according to the manufacturer's instruction. The culture medium was replaced after 6 hours. Cells were harvested for RNA extraction and protein extraction 48 hours after transfection.

### 2.3 Quantitative real‐time polymerase chain reaction (qRT‐PCR) assay

Total RNA in tissues and cells was extracted by TRIzol reagent (Invitrogen) and reagent Kit (TAKARA, Japan) according to the manufacturer's protocol. cDNA was reversed by PrimeScript TM RT reagent Kit (TAKARA, Japan). Quantitative real‐time PCR (qRT‐PCR) was performed on a 7300HT system (ABI) using SYBR Premix Ex Taq II kit (TAKARA, Japan). The internal control was set as glyceraldehyde‐3‐phosphate dehydrogenase (GAPDH) or snRNA U6. The relative expression level was calculated by 2^−ΔΔCt^ method. The primers were as follows: *MEG3* reverse 5'‐ACATTCGAGGTCCCCTTCCCACGTAGGCAT‐3' and forward 5'‐GGGCTTCTGGAATGAGCATGCTACTG‐3'; GAPDH reverse 5'‐GGATCTCGCTCCTGGAAGATG‐3' and forward 5'‐GCACCGTCAAGGCTGAGAAC‐3'; miR‐421 reverse 5'‐TATGGTTGTTCTGCTCTCTGTGTC‐3' and forward 5'‐CTCACTCACATCAACAGACATTAATT‐3'; U6 reverse 5'‐AACGCTTCACGAATTTGCGT‐3' and forward 5'‐CTCGCTTCGGCAGCACA‐3'.

### 2.4 Invasion assays

Transwell filter (8‐mm aperture; Millipore) was used to analyze the invasive ability of cells. The matrix and DMEM were prepared in the ratio of 1:6. The 8‐µm Millipore Transwell chamber was placed on a 24‐well plate and then we added 50‐µL solution into each chamber. Cells in a density of 1 × 10^5^ were inoculated in the serum‐free medium of 200 µL in the upper chamber, and the medium containing 500‐µL 10% FBS were placed in the lower chamber. After being incubated in the incubator at 37°C for 24 hours, the cells remaining in the upper room were gently removed with cotton swabs. Cells in the lower chamber were immobilized with methanol for 30 minutes, and then we stained them with crystal violet for 20 minutes.

### 2.5 Wound‐healing assay

Cells were cultured in a six‐well plate to reach 90%‐100% confluency. Cells were cultured in a six‐well plate to reach 90%‐100% confluency. Gently and slowly scratch the monolayer with a new 1‐mL pipette tip across the center of the well. While scratching across the surface of the well, always keep the long‐axial of the tip perpendicular to the bottom of the well. Photographs of the same region were taken with the same microscope, and scratch changes at different time points were recorded.

### 2.6 Cell proliferation

Cell Counting Kit 8 (CCK8) (Beyotime Jiangsu, China) was used to detect the cell proliferation capacity. Cells were inoculated into 96‐well plates till a density of 1 × 10^3^ cells per well. Ten microliter of Cell Counting Kit 8 reagent was added into each well at 0, 12, 24, 48, and 72 hours. The plates were placed for a subsequent 2‐hour incubation at 37°C. The absorbance was measured at the wavelength of 450 nm according to the manufacturer's instructions.

### 2.7 Western blotting analysis

Cells were harvested on ice in RIPA buffer (50 mmol/L Tris, pH 7.4, 150 mmol/L NaCl, 0.1% SDS, 0.5% sodium deoxycholate, and 1.0% NP‐40) with protease inhibitor cocktail (Roche). The membrane was blocked with 5% BSA for 2 hours and performed incubation with specific primary antibodies overnight at 4˚C, including E‐cadherin (1:500, ab76055, abcam, USA); Vimentin (1:1000, CST5741, CST, USA); ZEB2 (1:1000, GTX129243, GeneTex, USA); GAPDH (1:1000, ab181602, abcam). The membrane was then incubated with HRP Goat‐anti‐Rabbit (1:2000) or Goat‐anti‐Mouse (1:2000) for 2 hours at room temperature. The strip images analysis was performed by ImageJ and Prism 8 Software.

### 2.8 Luciferase reporter assay

The wild‐type GV272‐*MEG3* and the mutant plasmids were co‐transfected into 293T cells cultured in 24‐well plates, and 1‐µL Lipofectamine 2000 was added into each well referring to the manufacturer's protocol. Cells were harvested at 48 hours after transfection, and luciferase activities were analyzed through the Luc‐PairTM Duo‐Luciferase Assay Kit 2.0 (Genecopoeia, USA).

### 2.9 Statistical analysis

Statistical analysis was accomplished using GraphPad Prism 7.0 (GraphPad Software). All data are presented as mean ± SD. All statistical analyses provided contain only two groups. Differences were analyzed by two‐tailed Student's *t* test and *P* < .05 was considered significant.

## 3 RESULTS

### 3.1 The expression of *MEG3* in HNSCC tissues and cell lines

**FIGURE 1:**
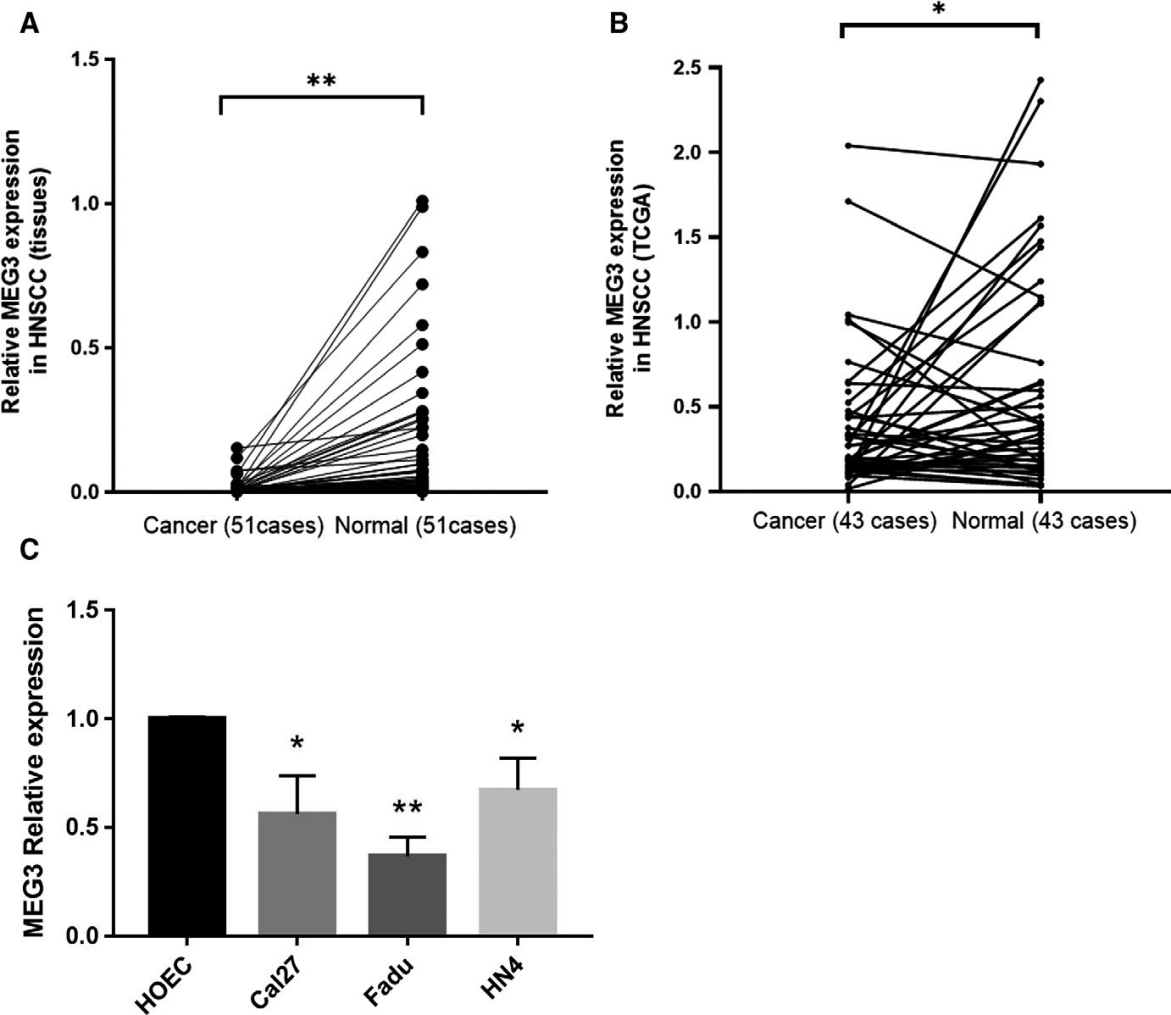
A, The relative expression of *MEG3* in 51 cases of HNSCC tissues and adjacent normal tissues in pairs. B, The relative expression of *MEG3* in TCGA database. C, The relative expression of *MEG3* in HNSCC cell lines Cal27, Fadu, and HN4 and oral epithelium cell (HOEC). *P < .05, **P < .01

Lower expression of *MEG3* in HNSCC has been verified in cancer tissues and cell lines. qRT‐PCR was performed in 51 cases of HNSCC tissues, and the results showed that the expression of *MEG3* was significantly lower compared to the corresponding adjacent normal tissues (Figure 1A). In The Cancer Genome Atlas (TCGA), significant difference appeared in 43 samples of HNSCC cancer and adjacent normal tissues in pairs (Figure 1B). In cell lines Cal27, Fadu and HN4, and HOEC, the results confirmed that compared with normal oral epithelial cells, the expression of *MEG3* in HNSCC cells was downregulated (Figure 1C).

### 3.2 *MEG3* significantly inhibited cell malignant phenotype in HNSCC

**FIGURE 2:**
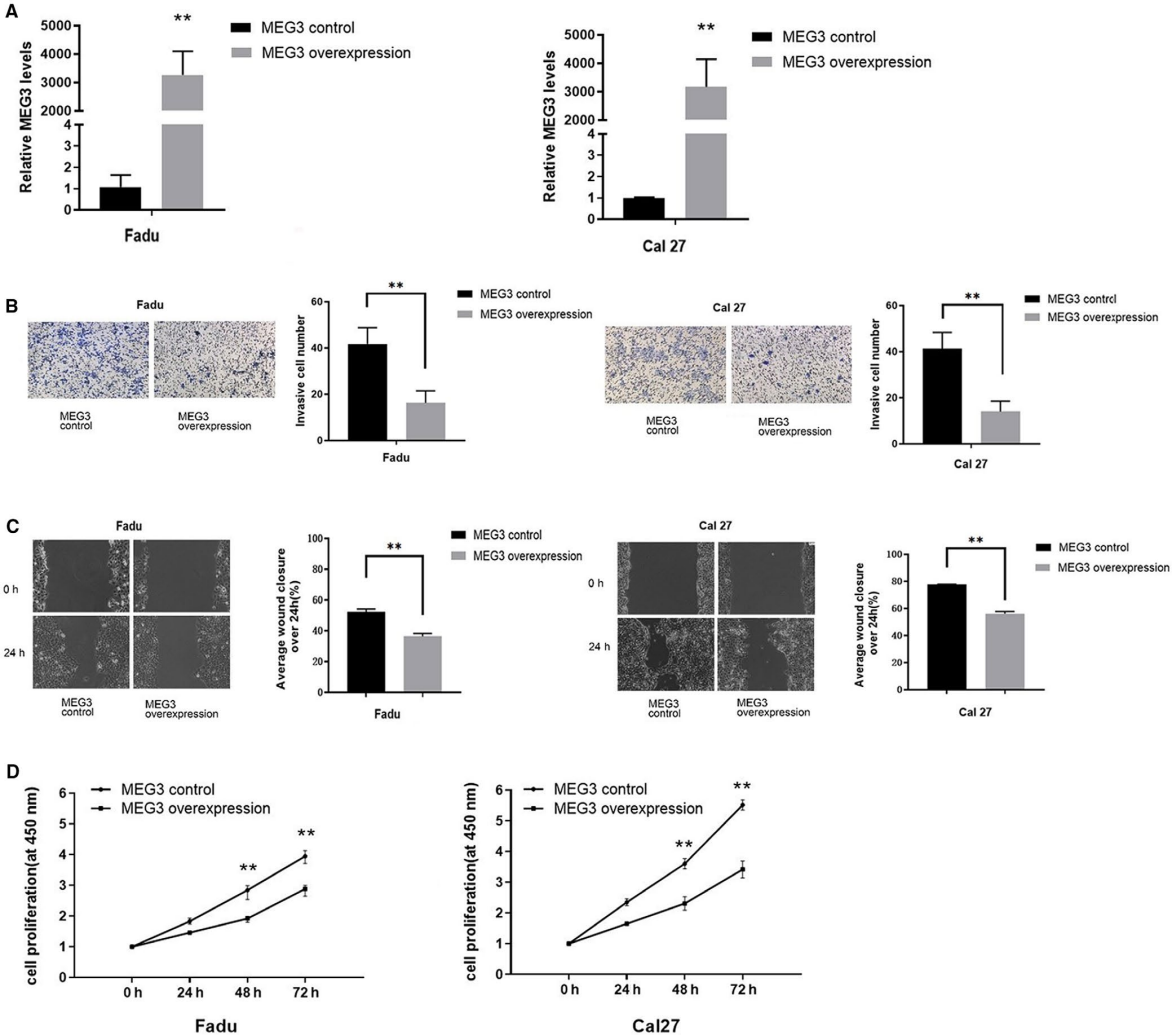
A, The relative expression of *MEG3* in Fadu and Cal27 cells transfected with empty vectors (*MEG3* control) or *MEG3*‐overexpressed lentivirus (*MEG3* overexpression). B, The invasion ability of transfected Fadu and Cal27 cells. C, The migration of transfected Fadu and Cal27 cells. D, The proliferation of transfected Fadu and Cal27 cells. *P < .05, **P < .01

*MEG3*‐overexpressed lentivirus was transfected into Cal27 and Fadu cells (Figure 2A). The results indicated that *MEG3* was significantly upregulated. In order to evaluate the *MEG3*’s regulatory roles on the phenotype of HNSCC cells, we conducted transwell, wound healing, and CCK8 assays. The invasion (Figure 2B), migration (Figure 2C) and proliferation (Figure 2D) activities of HNSCC cells decreased significantly.

### 3.3 *MEG3* significantly inhibited cell EMT in HNSCC

**FIGURE 3:**
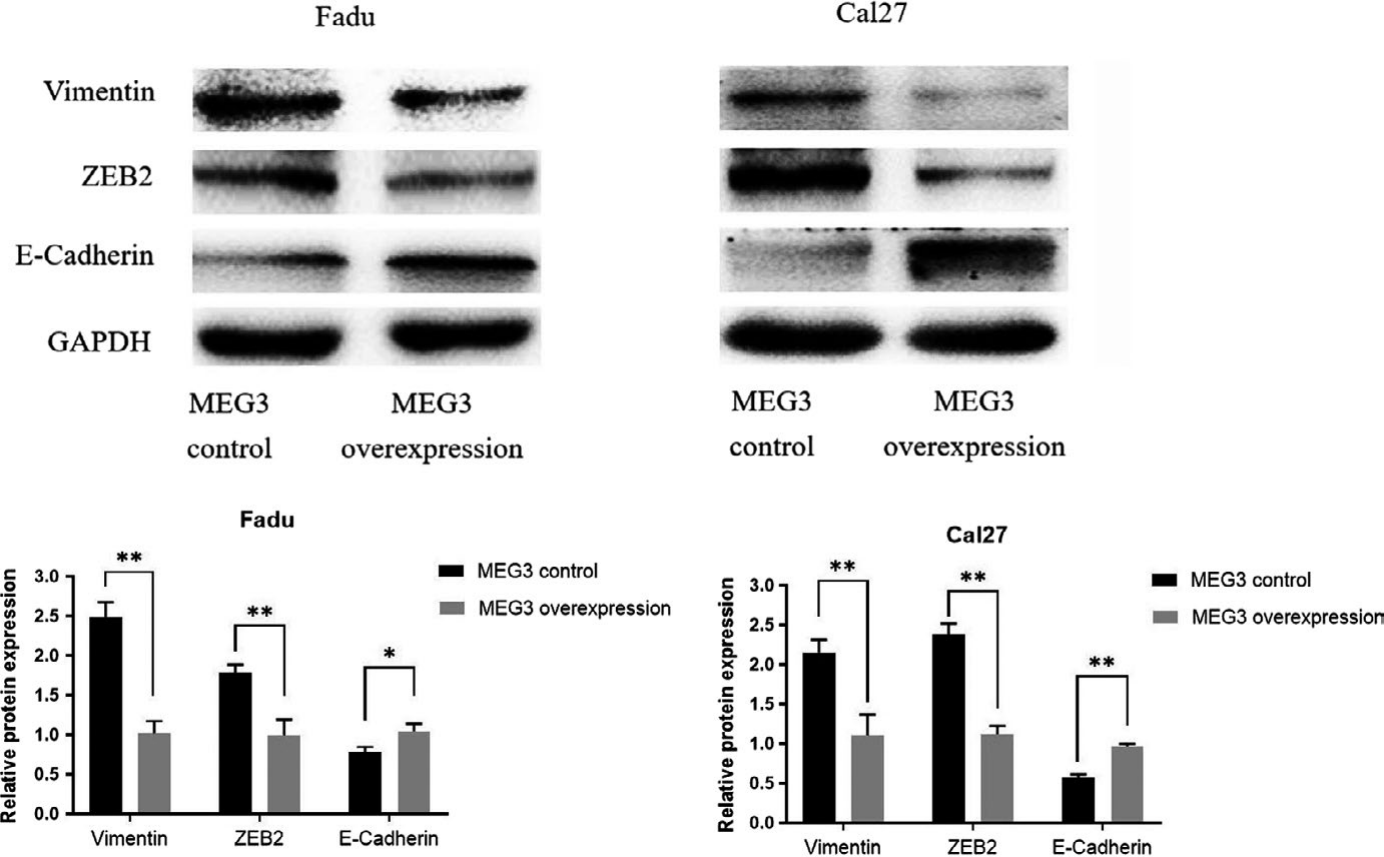
*MEG3* inhibited the EMT process. The relative protein levels of Vimentin, ZEB2, and E‐cadherin in transfected Fadu and Cal27 cells. *P < .05, **P < .01

We detected the effects of overexpression *MEG3* RNA on protein levels in the EMT process, including ZEB2, Vimentin, and E‐cadherin. We found that E‐cadherin expression levels increased, while ZEB2 and Vimentin decreased significantly in *MEG3*‐transfected cells (Figure 3). These results indicated that upregulation of the expression level of *MEG3* in HNSCC inhibited the EMT process of cells.

### 3.4 MiR‐421 was a target gene of *MEG3* in HNSCC cells

**FIGURE 4:**
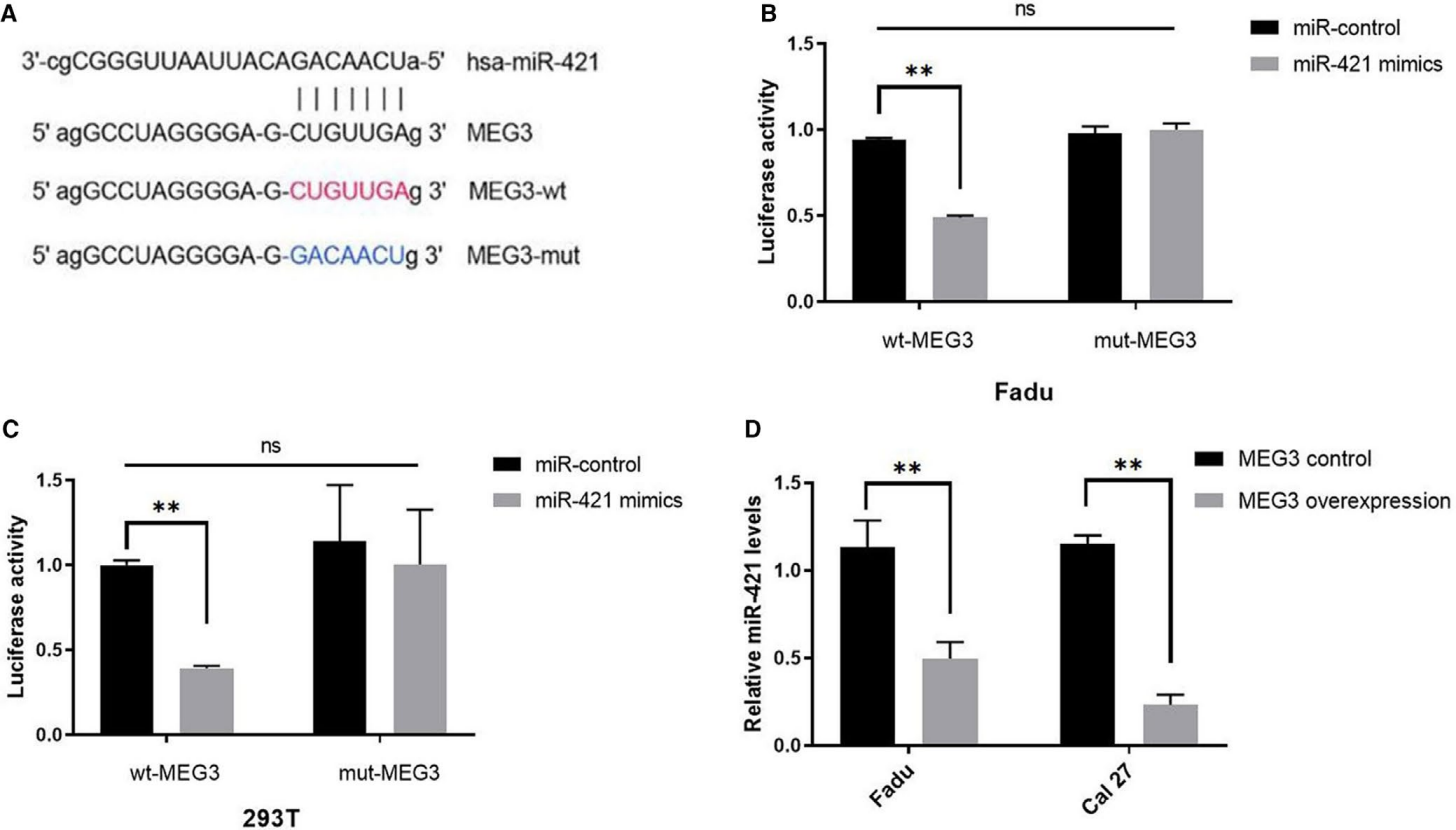
A, *MEG3* wild‐type and mutant plasmid. B and C, Luciferase reporter assays in 293T and Fadu cells. MiR‐421 was a target of *MEG3* in HNSCC. D, The relative expression of miR‐421 in transfected Fadu and Cal27 cells. *P < .05, **P < .01

MiR‐421 was predicted to be a potential target for *MEG3. MEG3* wild‐type and mutant plasmid were constructed based on the miR‐421 and *MEG3* binding sequences. (Figure 4A). Plasmids expressing *MEG3* or miR‐421 and control empty vectors were co‐transfected into 293T and Fadu cells. The results illustrated that the luciferase activity was decreased in the miR‐421 mimics and the *MEG3*‐wt co‐transfected group (Figure 4B,C). The results of qRT‐PCR showed that *MEG3* suppressed the expression of miR‐421 (Figure 4D).

### 3.5 MiR‐421 promoted cell invasion and proliferation in HNSCC cell lines

**FIGURE 5:**
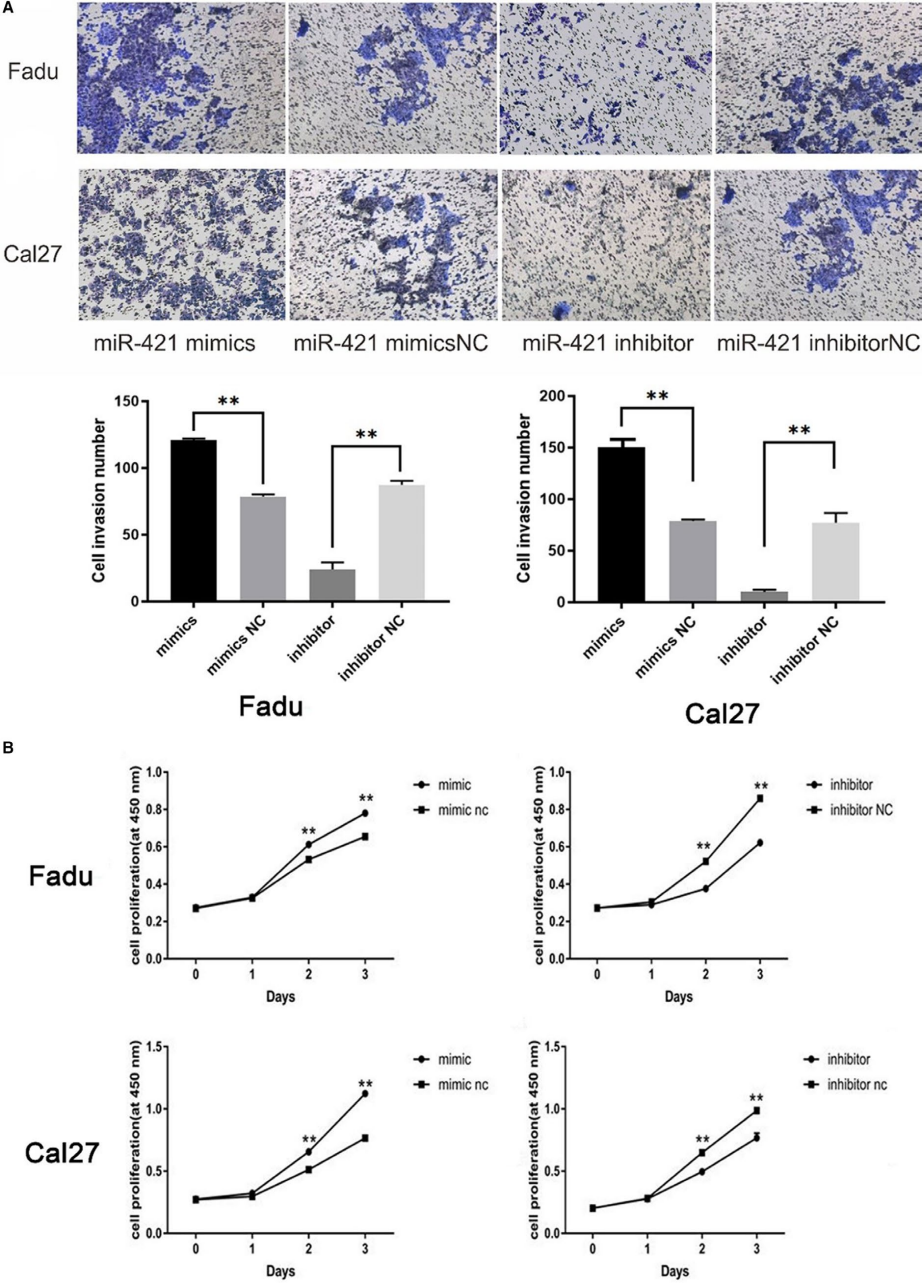
A, The invasion ability of Fadu and Cal27 cells transfected with miR‐421 mimics or inhibitors. B, The proliferation ability of transfected Fadu and Cal27 cells. *P < .05, **P < .01

To assess the effect of miR‐421 on invasion and proliferation in HNSCC, miR‐421 mimics or inhibitor plasmids were transfected into Fadu and Cal27 cells. Transwell (Figure 5A) and CCK8 (Figure 5B) assays were used to detect the cell invasion and proliferation ability. In miR‐421 mimics group, miR‐421 RNA was overexpressed, and the invasion and proliferation activities were significantly increased compared with the miR‐421 inhibitor groups. Our experimental data indicated that miR‐421 increased the invasion and proliferation in HNSCC cells.

### 3.6 E‐cadherin was a target gene of miR‐421 in HNSCC cells

**FIGURE 6:**
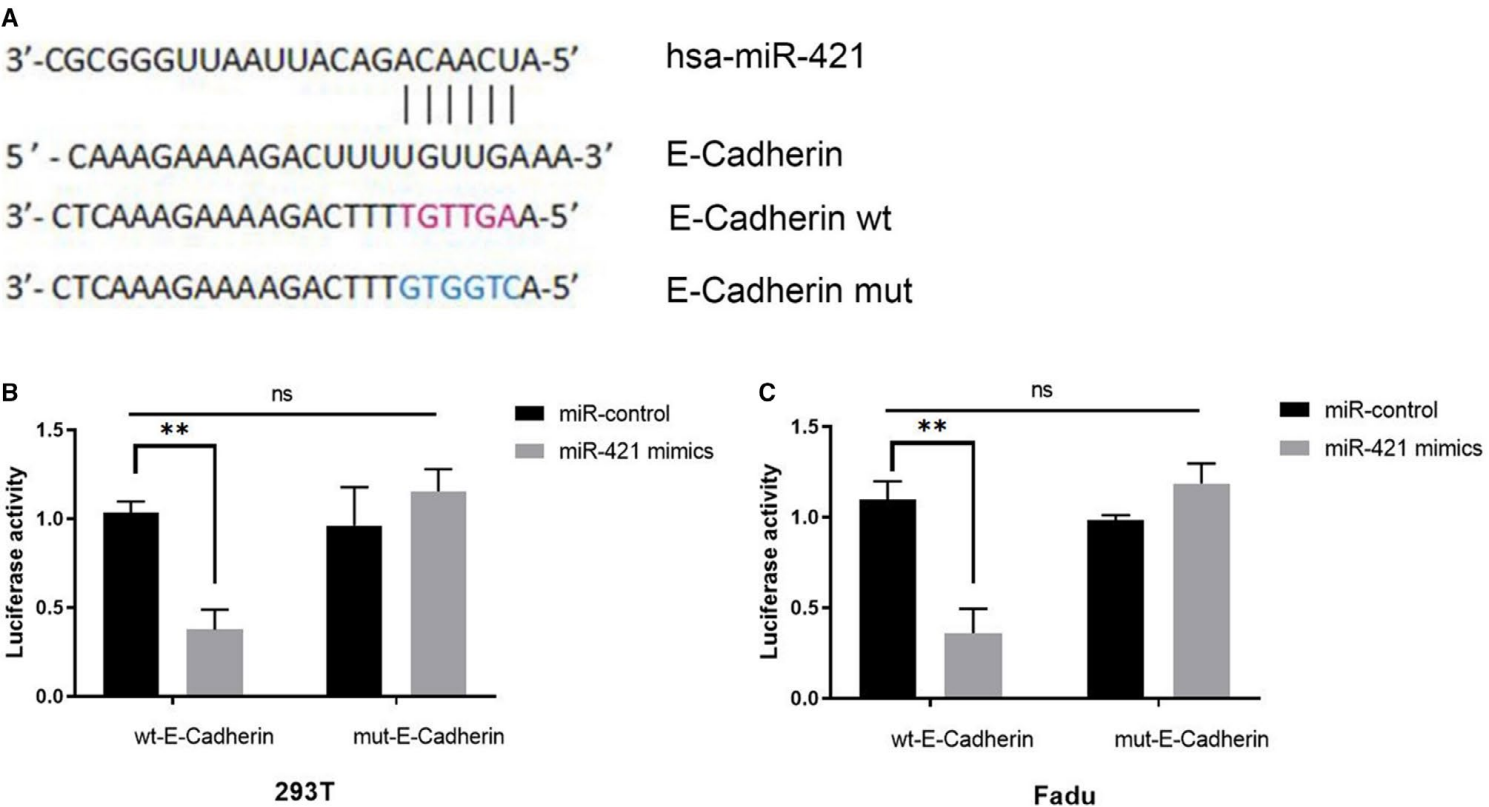
A, E‐cadherin wild‐type and mutant plasmids. B and C, Luciferase reporter assays in 293T and Fadu cells. *P < .05, **P < .01

Through miRanda software analysis, E‐cadherin was found to be the possible target gene of miR‐421. The GV272‐E‐cadherin wild‐type and GV272‐E‐cadherin mutant vector (Figure 6A) were constructed and co‐transfected into 293T and Fadu cells. Together, miR‐421 mimics and miR‐control were co‐transfected, respectively. Results demonstrated that the luciferase activity decreased in the miR‐421 mimics and the wt‐E‐cadherin co‐transfected group (Figure 6B,C). These results indicated that E‐cadherin was a target gene of miR‐421.

### 3.7 *MEG3* negatively interacting with miR‐421 regulated E‐cadherin expression in HNSCC cells

**FIGURE 7:**
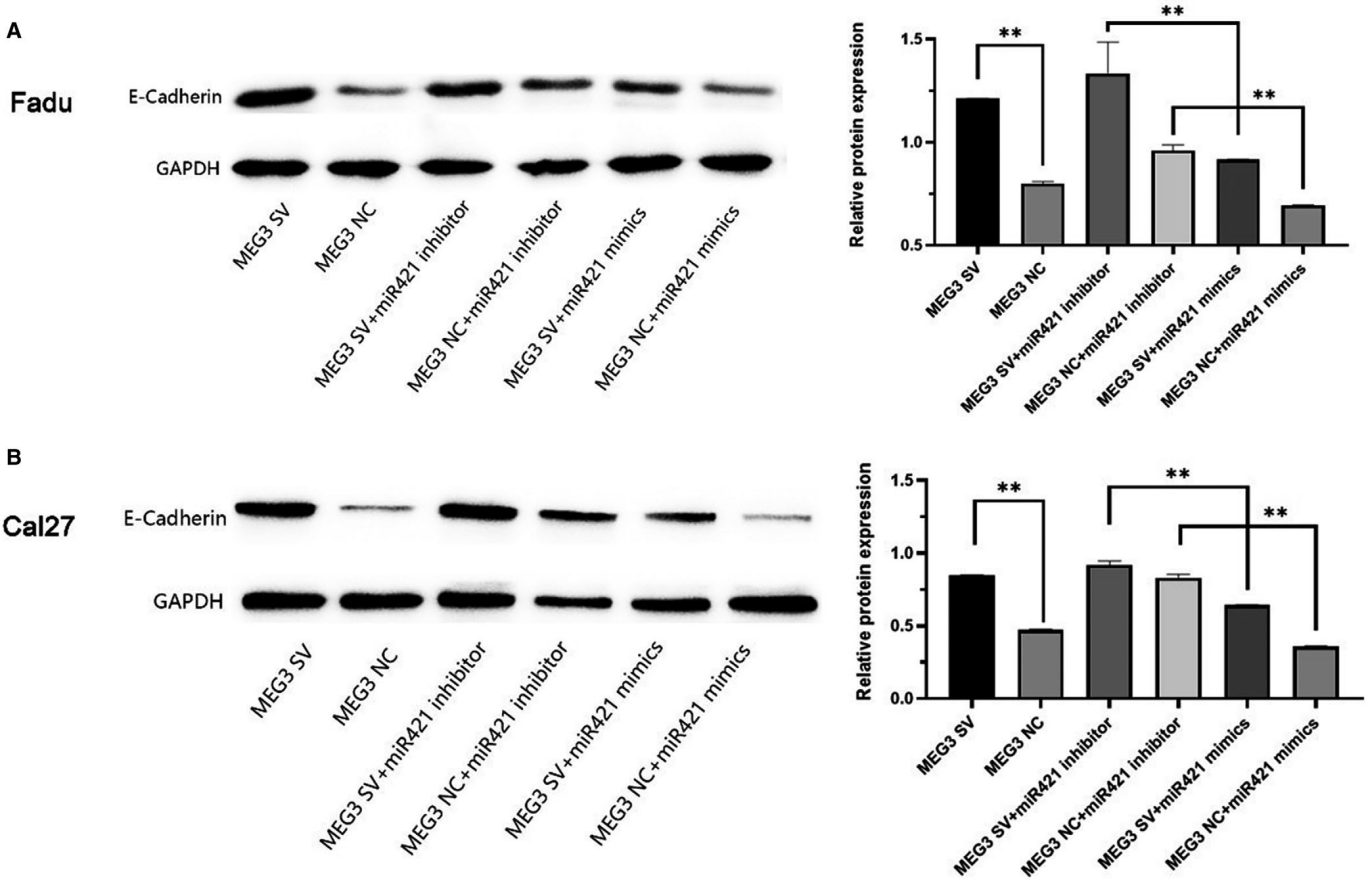
A and B, Relative E‐cadherin expression in transfected Fadu and Cal27 cells. **P < .01, *P < .05

*MEG3* was presumed to upregulate E‐cadherin through sponging miR‐421. Upregulating *MEG3* promoted the expression of E‐cadherin, but co‐transfection of miR‐421 mimics offset the effect in Fadu (Figure 7A) and Cal27 cells (Figure 7B). Co‐transfection of miR‐421 inhibitor increased this effect. Therefore, our results demonstrated that *MEG3* regulates E‐cadherin expression by interacting with miR‐421 in HNSCC cells.

## 4 DISCUSSION

LncRNA is a heterogeneous group of ncRNAs, more than 200 nucleotides long, without protein coding ability, and earlier, it has been considered as a kind of transcriptional noise. Recent studies indicated that it was linked to all six hallmarks of cancer including activating invasion, evading growth suppressors, resisting apoptosis, continuous growth‐promoting signaling, enabling continuous proliferation even at limited nutrient conditions, and metastasis and inducing angiogenesis.^5^ LncRNA can regulate the occurrence and development of cancer through a variety of mechanisms. At present, the research of lncRNA has become the frontier of human cancer research.^6^, ^25^

*MEG3* is a lncRNA located on chromosome 14q32, which is closely related to the occurrence and development of cancer.^13^, ^14^ Earlier studies reported that *MEG3* expression was abnormally low or even absent in various cancer tissues and cancer cell lines.^26^ Inactivation of the *MEG3* gene in these cancers is partly attributed to promoter silencing by hypermethylation and contributes to cancer development. For example, the expression of *MEG3* in gallbladder carcinoma tissues is significantly lower than that in normal tissues.^8^ In lung carcinoma, downregulation of *MEG3* enhances cisplatin resistance through the WNT/β‐catenin pathway activation.^15^ Our results were in line with these studies.

The competitive endogenous RNA (ceRNA) hypothesis suggests that lncRNAs and miRNAs compete for posttranscriptional control through sponge interactions to influence cancer development. The interaction of lncRNA, miRNA, and target genes in cancer pathogenesis is a new field of investigation in cancerigenesis.^7^, ^27^ In recent years, many miRNAs have been discovered as oncogenes or cancer suppressor genes, and they are involved in the development of various malignant cancer. Decreased *MEG3* expression was identified in cervical cancer and thus upregulated the cell proliferation ability by sponging miR‐21.^16^ *MEG3* can inhibit the growth of lymphoblastic lymphoma through the miRNA‐214/AIFM2 axis.^28^ In this study, dual luciferase assay verified that miR‐421 binding to E‐cadherin. miR‐421 was reported to overexpress in many cancers and had been associated with poor prognosis. The tested results of qRT‐PCR showed that miR‐421 was negatively regulated by *MEG3* in HNSCC cells. *MEG3* regulated E‐cadherin by sponging miR‐421.

E‐cadherin is one of the most important marker proteins in the process of EMT, which is closely related to the invasion of cancer. Increased E‐cadherin combined with decreased ZEB2 and Vimentin indicates well‐circumscribed, nonaggressive, low metastatic potential.

It had been found that *MEG3* regulated the expression of E‐cadherin, a downstream target gene, and regulated the EMT process of HNSCC by regulating the expression of miR‐421. It was a limitation of our research, since we carried out our research without mouse models. We will further the study into the mechanism and add verification in vivo experiment. This discovery provided a new idea for the study of HNSCC and a possible therapeutic target for its clinical treatment.

## 5 CONCLUSIONS

The low expression of *MEG3* in HNSCC tissues and cell lines suggested that *MEG3* may be a potential biomarker of HNSCC. After overexpression of *MEG3*, the migration, proliferation, and invasion of HNSCC cells were significantly inhibited. Further studies found that *MEG3* inhibited the EMT process by targeting E‐cadherin and decreasing the expression of miR‐421.

## CONFLICT OF INTEREST

None.

## AUTHOR CONTRIBUTIONS

HY designed the research, supervised the study, and helped in drafting the manuscript. YJ, GF, YH, YY, and RW were involved in the acquisition of the specimen, performed the experiments and analysis of data. YJ and GF wrote the manuscript. All authors were involved in evaluating the manuscript. All authors approved the submitted version.

## Data Availability

The data that support the findings of this study are available from the corresponding author upon reasonable request.
